# Reactive Oxygen Species Downregulate Transient Receptor Potential Melastatin 6 Expression Mediated by the Elevation of miR-24-3p in Renal Tubular Epithelial Cells

**DOI:** 10.3390/cells10081893

**Published:** 2021-07-26

**Authors:** Chieko Hirota, Yui Takashina, Yuta Yoshino, Hajime Hasegawa, Ema Okamoto, Toshiyuki Matsunaga, Akira Ikari

**Affiliations:** 1 Laboratory of Biochemistry, Department of Biopharmaceutical Sciences, Gifu Pharmaceutical University, Gifu 501-1196, Japan; 165067@gifu-pu.ac.jp (C.H.); 145037@gifu-pu.ac.jp (Y.T.); yoshino-yu@gifu-pu.ac.jp (Y.Y.); 185020@gifu-pu.ac.jp (E.O.); 2 Saitama Medical Center, Department of Nephrology and Hypertension, Saitama Medical University, Saitama 350-8550, Japan; hase2126@saitama-med.ac.jp; 3 Education Center of Green Pharmaceutical Sciences, Gifu Pharmaceutical University, Gifu 502-8585, Japan; matsunagat@gifu-pu.ac.jp

**Keywords:** type 2 diabetes mellites, glycated albumin, hypomagnesemia, miRNA, TRPM6

## Abstract

Background: A low level of serum magnesium ion (Mg^2+^) is associated with type 2 diabetes mellitus (T2D). However, the molecular mechanism of Mg^2+^ deficiency has not been fully clarified. The current study sought to assesses the effect of reactive oxygen species on the expression of Mg^2+^ channels and miRNA. Methods: The expression of Mg^2+^ channels and miRNA were examined by real-time polymerase chain reaction. Intracellular Mg^2+^ concentration was measured by Magnesium Green fluorescence measurement. Results: The mRNA level of transient receptor potential melastatin 6 (TRPM6), which functions as Mg^2+^ influx channel in the distal convoluted tubule (DCT) of the kidney, was decreased by glycated albumin (GA), but not by insulin in rat renal tubule-derived NRK-52E cells. The mRNA levels of TRPM7, a homologue of TRPM6, and CNNM2, a Mg^2+^ efflux transporter located at the basolateral membrane of DCT, were changed by neither GA nor insulin. The generation of reactive oxygen species (ROS) was increased by GA. Hydrogen peroxide (H_2_O_2_) dose-dependently decreased TRPM6 mRNA, but it inversely increased the reporter activity of TRPM6. H_2_O_2_ accelerated the degradation of TRPM6 mRNA in actinomycin D assay without affecting TRPM7 and CNNM2 mRNA expressions. Nine miRNAs were considered as candidates for the regulator of stability of TRPM6 mRNA. Among them, miR-24-3p expression was increased by H_2_O_2_. The H_2_O_2_-induced reduction of TRPM6 mRNA was rescued by miR-24-3p siRNA. Magnesium Green fluorescence measurement showed that Mg^2+^ influx is suppressed by H_2_O_2_, which was rescued by an antioxidant and miR-24-3p siRNA. Conclusions: We suggest that GA decreases TRPM6 expression mediated by the elevation of ROS and miR-24-3p in renal tubular epithelial cells of T2D.

## 1. Introduction

The body content of magnesium is controlled by the absorption in the intestine and reabsorption in the kidney. The concentration of serum magnesium ion (Mg^2+^) is maintained within narrow range from 0.75 to 0.95 mmol/L under physiological conditions [1]. Approximately 80% of the total serum Mg^2+^ is filtered in the glomeruli of kidney with more than 95% is reabsorbed along the nephron, and only 5% is excreted into the urine [2]. The majority of filtrated Mg^2+^ is reabsorbed via a paracellular route in the thick ascending limb of the Henle’s loop. The reabsorption rate is only 5% in the distal convoluted tubule (DCT), but this process plays an important role in the fine-tuning of urinary Mg^2+^ excretion. Transient receptor potential melastatin 6 (TRPM6) is located at the apical membrane of DCT, a main site of active renal Mg^2+^ reabsorption [3], and may facilitate Mg^2+^ influx from the lumen to the cells. CNNM2, a Mg^2+^ efflux transporter located at the basolateral membrane, is considered to transport Mg^2+^ from the cells to the blood in the DCT, but it is controversial [4,5].

Many investigators reported that Mg^2+^ deficiency may be one of the important risk factors for lifestyle-related diseases such as type 2 diabetes mellitus (T2D), hypertension, and hyperlipidemia [6,7]. High Mg^2+^ intake is associated with a reduced incidence of T2D in women [8]. T2D patients usually have impaired sensitivity to insulin, so-called insulin resistance, and often develop a hyperglycemic condition and nephropathy [9]. A clinical randomized double-blind placebo-controlled trial shows the administration of Mg^2+^ supplementation improves insulin resistance and metabolic control in T2D patients [10]. Thus, there is no doubt about the correlation between hypomagnesemia and T2D. Takayanagi et al. [11] reported that the reduction of TRPM6 expression in obese T2D rats may cause hypermagnesiuria and nephropathy. However, it is unknown how TRPM6 expression is downregulated in T2D.

Despite the importance of TRPM6 in the maintenance of Mg^2+^ homeostasis, the regulatory mechanism of TRPM6 gene is mostly unknown. So far, we revealed that epidermal growth factor (EGF) increases the transcriptional activity of TRPM6 mediated through the activation of a mitogen-activated protein kinase kinase (MEK) and extracellular signal-regulated kinase (ERK), so-called MEK/ERK signaling pathway using rat renal tubular NRK-52E and human embryonic kidney 293 (HEK293) cells [12,13]. Renal Mg^2+^ wasting is an undesirable side effect during anticancer therapy. EGF receptor (EGFR) tyrosine kinase inhibitors and EGFR-targeting monoclonal antibody may develop hypomagnesemia mediated through the inhibition of MEK/ERK pathway and the reduction of TRPM6 expression [14]. In addition, cisplatin, a platinum anticancer drug, decreases the mRNA levels of EGF and TRPM6 in the rat kidney [15]. Therefore, the MEK/ERK pathway may play a crucial role in the regulation of TRPM6 mRNA expression and Mg^2+^ homeostasis under physiological conditions.

The production of advanced glycation end-products (AGEs) are implicated in the development of diabetic nephropathy [16]. The activity of TRPM6 channel has been reported to be inhibited by hydrogen peroxide (H_2_O_2_) in HEK293 transfected with human TPRM6 [17]. In contrast, the effect of oxidative stress on TRPM6 expression remains to be elucidated. In the present study, we found that TRPM6 expression is decreased by glycated albumin (GA) and H_2_O_2_ in NRK-52E cells. To clarify the regulatory mechanism of TRPM6 expression, transcriptional activity, mRNA stability, and Mg^2+^ flux of TRPM6 were investigated using the dual-luciferase reporter assay, actinomycin D assay, and fluorescence assay, respectively.

## 2. Materials and Methods

### 2.1. Materials

GA, insulin, and N-acetyl-l-cysteine (NAC) were obtained from Sigma-Aldrich (St. Louis, MO, USA) and dissolved in water. 2′,7′-dichlorodihydrofluorescein diacetate (H_2_DCFDA) and Mag-Fura-2 AM were from Thermo Fisher Scientific (Waltham, MA, USA).

### 2.2. Cell Culture and Transfection

Normal rat renal tubule-derived NRK-52E cells (IFO50480) were purchased from Japanese Collection of Research Biosciences (Osaka, Japan). So far, we reported that mRNAs and proteins of TRPM6, TRPM7, and CNNM2 were detected in NRK-52E cells by real time PCR and Western blotting, respectively [18]. The cells were cultured in Dulbecco’s modified Eagle’s medium supplemented with 5% fetal bovine serum as described previously [13]. Universal negative control and miR-24-3p siRNAs (Sigma-Aldrich) were transfected with Lipofectamine RNAiMAX (Thermo Fisher) according to manufacturer’s instructions.

### 2.3. Isolation of Total RNA and Quantification of mRNA

Total RNA was isolated form NRK-52E cells with TRI reagent (Molecular Research Center, Cincinnati, OH, USA) and reverse transcriptional reaction was done using ReverTra Ace qPCR RT Kit (Toyobo Life Science, Osaka, Japan). For the assay of miRNA, cDNA was prepared using Mir-X miRNA First-Strand Synthesis Kit (Takara Bio, Shiga, Japan). Quantitative real-time polymerase chain reaction (PCR) were done using a, Eco Real-Time PCR system (AS One, Osaka, Japan) with a THUNDERBIRD SYBR qPCR Mix (Toyobo Life Science). Primer pairs used for PCR are shown in Table 1. After subtracting the threshold cycle (Ct) obtained for β-actin, the relative change of mRNA expression was calculated by ΔΔCt method.

### 2.4. Preparation of Cytoplasmic Extracts and Western Blotting

The preparation of cytoplasmic extracts and sodium dodecyl sulfate polyacrylamide gel electrophoresis were carried out as described previously [13]. After blotting samples to poly (vinylidene fluoride) membrane, the membrane was blocked with a 2% bovine serum albumin for 30 min. Then, the membrane was incubated with anti-p-ERK and anti-ERK antibodies (1:1000 dilution) at 4 °C for 16 h, followed by a peroxidase-conjugated secondary antibody (1:3000 dilution) at room temperature for 1.5 h. Finally, the chemiluminescence of horseradish peroxidase was detected using a C-DiGit Blot Scanner (LI-COR Biotechnology, Lincoln, NE, USA). The band density was quantified using ImageJ software (National Institute of Health software). β-actin was used for normalization.

### 2.5. Luciferase Reporter Assay

So far, we constructed the luciferase reporter vector containing promoter of the human *TRPM6* gene [19]. The reporter vectors of mock and TRPM6 were transfected into the cells with HilyMax (Dojindo Laboratories, Kumamoto, Japan). A pRL-TK vector (Promega, Madison, WI, USA), which contains *Renilla* luciferase gene under the herpes simplex virus thymidine kinase promoter, was used for normalizing transfection efficiency. The Dual-Glo Luciferase Assay System (Promega) was used to explore the activities of firefly and renilla luciferases.

### 2.6. Mg^2+^ Influx Assay

The change in intracellular free Mg^2+^ concentration ([Mg^2+^]_i_) was measured with a Mg^2+^-sensitive fluorescent dye, Magnesium Green AM (Thermo Fisher Scientific). NRK-52E cells cultured on 96 well plates were loaded with Hank’s Balanced salt solution (HBSS) containing 2 μM Magnesium Green AM at 37 °C for 30 min, followed by washing with dye-free HBSS twice. After replacement of extracellular solution from nominally MgCl_2_-free HBSS to 5 mM MgCl_2_-containing HBSS at 0 min, the fluorescence intensity of Magnesium Green was measured every 20 s at 485 nm (excitation)/535 nm (emission) using an Infinite F200 Pro fluorescence microplate reader (Tecan, Mannedorf, Switzerland). The change of fluorescence intensity of Magnesium Green was represented as a percentage at 0 min.

### 2.7. Reactive Oxygen Species (ROS) Generation

H_2_DCFDA, a marker of a wide spectrum of ROS, was used for the measurement of generation of intracellular ROS. NRK-52E cells were incubated in the presence or absence of insulin and 500 μg/mL GA for 1 h. Then, the cells were incubated with 10 μM H_2_DCFDA at 37 °C for 30 min. After washing with HBSS twice, the fluorescence intensity of DCF was measured by fluorescence microplate reader.

### 2.8. Statistical Analysis

All data are presented as mean ± standard error using KaleidaGraph software (Synergy Software, Reading, PA, USA). Statistical significance was evaluated by a one-way ANOVA analysis for multiple comparison between groups. Statistical comparisons between two groups were conducted by a Student’s *t*-test. A *p* value of <0.05 was taken as significant.

## 3. Results and Discussion

### 3.1. Decrease in TRPM6 Expression in NRK-52E Cells by GA

**Figure 1:**
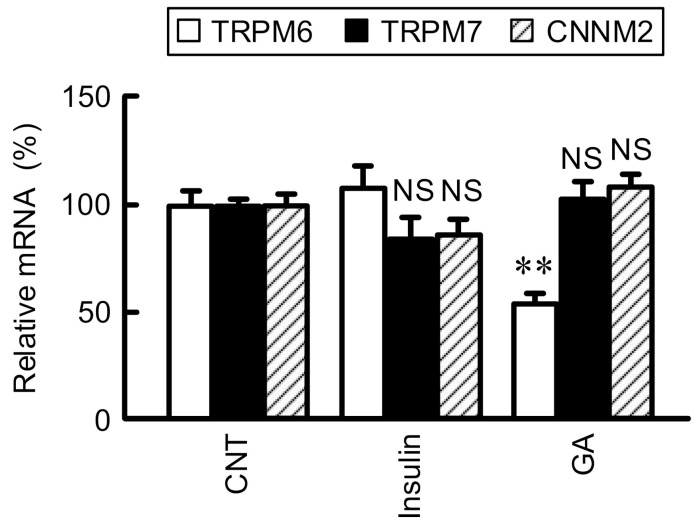
Effects of insulin and GA on the expression of Mg^2+^ transporters. The cDNA was prepared from total RNA of NRK-52E cells treated with vehicle (CNT), 10 nM insulin, or 500 μg/mL GA for 6 h. Real time PCR was carried out by primers for TRPM6, TRPM7, CNNM2, and β-actin. The expression levels of TRPM6, TRPM7, and CNNM2 were normalized by β-actin, and were represented as percentage of CNT. *n* = 3–4. ** *p* < 0.01 and ^NS^ *p* > 0.05 compared with CNT.

TRPM6 and CNNM2 are localized at the apical and basolateral membranes of DCT, respectively, and may be involved in the transcellular reabsorption of Mg^2+^ [4,20]. In contrast, TRPM7, a close homologue of TRPM6, is ubiquitously found in most tissues and involved in the regulation of Mg^2+^ homeostasis [21]. In a nested case-control study of the Women’s Health Study, two common non-synonymous variants of TRPM6 coding region, I1393V and K1584E polymorphisms, confer susceptibility to T2D in women with low Mg^2+^ intake [22]. The abnormal expression of TRPM6 may be involved in T2D, but the regulatory mechanism of TRPM6 expression is not fully understood. *Lepr^fa^* rats show hyperinsulinemia and staining of AGEs in the renal cortex [23]. Therefore, we investigated the effects of insulin and GA on TRPM6 expression using NRK-52E cells because TRPM6 is functionally expressed in the cells [12]. The mRNA level of TRPM6 was significantly decreased by GA, but not by insulin (Figure 1). In contrast, neither those of TRPM7 nor CNNM2 were changed by GA nor insulin. These results suggest that the elevation of GA concentration induces the decrease in TRPM6 expression in the kidney of T2D patients. Insulin stimulates TRPM6 activity mediated by increased phosphorylation of intracellular signaling cascade including phosphatidylinositol 3-kinase, Akt, and Rac1 [24], but Mg^2+^ reabsorption may be diminished by the reduction of TRPM6 expression. The association of Mg^2+^ deficiency and insulin resistance has been reported in obese children [25] and in normal human subjects with low Mg^2+^ diets [26]. Disruption of Mg^2+^ homeostasis caused by reduction of TRPM6 expression may be involved in the exacerbation of glucose tolerance and insulin resistance.

### 3.2. Involvement of ROS in the GA-Induced Reduction of TRPM6 Expression

**Figure 2:**
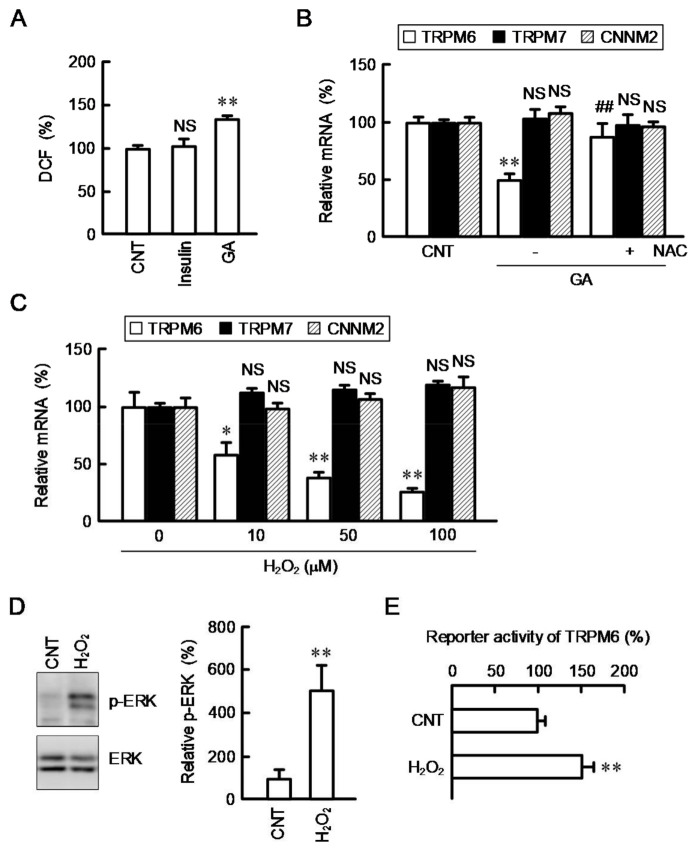
Effect of H_2_O_2_ on mRNA expression and reporter activity of TRPM6. (**A**) NRK-52E cells were treated with vehicle (CNT), 10 nM insulin, and 500 μg/mL GA for 1 h. The intracellular ROS contents were measured using DCF and represented as percentage of CNT. (**B**) Cells were treated in the presence or absence (CNT) of 500 μg/mL GA and 2 mM NAC for 6 h. The mRNA levels of TRPM6, TRPM7, and CNNM2 were represented as percentage of CNT. (**C**) Cells were treated with H_2_O_2_ for 6 h at the concentrations indicated. The mRNA levels of TRPM6, TRPM7, and CNNM2 were represented as percentage of 0 μM. (**D**) Cells were treated with vehicle (CNT) and 50 μM H_2_O_2_ for 1 h. The expression levels of p-ERK and ERK were measured by Western blotting and represented as percentage of CNT. (**E**) The reporter vector of TRPM6 was transfected into the cells using HilyMax. The transfection efficiency was corrected for by the ratio of firefly luciferase activity to *Renilla* luciferase activity and represented as percentage of CNT. *n* = 3–5. ** *p* < 0.01, * *p* < 0.05 and ^NS^ *p* > 0.05 compared with CNT or 0 μM. ^##^ *p* < 0.01 compared with -NAC.

AGEs have been reported to induce injury of renal tubular epithelial cells mediated by generation of ROS [27]. To clarify the involvement of ROS, we monitored intracellular ROS content using a fluorescent probe H_2_DCFDA. The fluorescence intensity of DCF was significantly increased by GA, but not by insulin (Figure 2A). The GA-induced reduction of TRPM6 expression was inhibited by NAC (Figure 2B). AGEs bind to the receptor of AGEs, leading to activation of its down-stream signaling and oxidative stress responses in renal cells [28]. We suggest that GA decreases TRPM6 expression mediated by the generation of ROS. To substantiate the involvement of ROS, the effect of hydrogen peroxide (H_2_O_2_), a ROS generator, on TRPM6, TRPM7, and CNNM2 expressions was investigated by real-time PCR. The mRNA level of TRPM6 was concentration-dependently decreased by H_2_O_2_ (Figure 2C). In contrast, the mRNA levels of TRPM7 and CNNM2 were not significantly changed. The mRNA expression of TRPM6 is upregulated by an activated MEK/ERK pathway in NRK-52E cells [13]. The involvement of inhibition of MEK/ERK pathway is hypothesized in the downregulation of TRPM6 expression by ROS. However, H_2_O_2_ increased the level of p-ERK without affecting total amount of ERK (Figure 2D). Furthermore, the reporter activity of TRPM6 was significantly exaggerated by H_2_O_2_ (Figure 2E). The p-ERK level is upregulated in the renal cortex of diabetic model mice [22]. So far, we reported that the reporter activity of TRPM6 is in proportion to the p-ERK level [18]. Therefore, there is no doubt that ROS can increase transcriptional activity of TRPM6. However, the mRNA level of TRPM6 was downregulated by H_2_O_2_ as shown in Figure 2C. We suggest that ROS can downregulates TRPM6 expression mediated by other mechanism, which acts more significantly than the transcriptional regulation. To clarify the hypothesis, we examined the effect of H_2_O_2_ on the mRNA stability of TRPM6.

### 3.3. Involvement of miR-24-3p in the ROS-Induced Reduction of TRPM6 Expression

**Figure 3:**
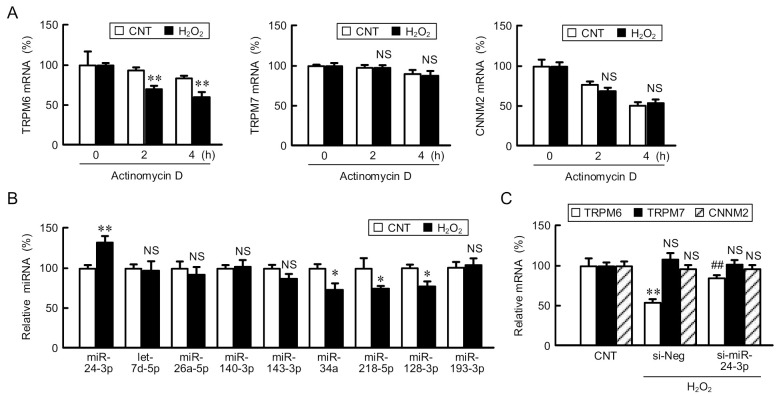
Involvement of miR-24-3p in the decrease in TRPM6 mRNA by H_2_O_2_. (**A**) NRK-52E cells were treated with actinomycin D in the presence or absence (CNT) of H_2_O_2_ for the periods indicated. The mRNA levels of TRPM6, TRPM7, and CNNM2 were represented as percentage of 0 h. (**B**) Cells were treated in the presence or absence (CNT) of H_2_O_2_ for 6 h. The miRNA levels were determined by real time PCR and compared with CNT. (**C**) The cells transfected with negative (si-Neg) or miR-24-3p siRNA (si-miR-24-3p) were treated with H_2_O_2_ for 6 h. The mRNA levels of TRPM6, TRPM7, and CNNM2 were represented as percentage of CNT. *n* = 3–4. ** *p* < 0.01, * *p* < 0.05 and ^NS^ *p* > 0.05 compared with CNT or 0 μM. ^##^ *p* < 0.01 compared with si-Neg.

In the mRNA stability assay, NRK-52E cells were treated with actinomycin D to prevent mRNA transcription. The mRNA level of TRPM6 was deteriorated depending on actinomycin D-treated period, which was reinforced by H_2_O_2_ (Figure 3A). In contrast, those of TRPM7 and CNNM2 were unchanged by H_2_O_2_. The mRNA stability of various genes is regulated by miRNA, RNA-binding proteins, and long non-coding RNAs [29]. The regulatory miRNAs of TRPM6 have been predicted using miRanda database [30], suggesting that hsa-let-7b, 7c, 7d, 7g, and 7f-1 can bind to 3′-untranslated region (3′UTR) of TRPM6 mRNA in colon adenocarcinoma. In the present study, we found other candidates including miR-24-3p, 26a-5p, 140-3p, 143-3p, 34a, 218-5p, 128-3p, 193-3p, and let-7d-5p interact with 3′UTR using Target Scan Program [31]. Among them, the expression level of miR-24-3p was significantly upregulated by H_2_O_2_, whereas those of other miRNAs were constant or decreased (Figure 3B). Therefore, we decided to investigate the involvement of miR-24-3p. The H_2_O_2_-induced reduction of TRPM6 mRNA was rescued by miR-24-3p siRNA (Figure 3C). In contrast, the mRNA levels of TRPM7 and CNNM2 were unchanged by H_2_O_2_ and miR-24-3p siRNA. We suggest that miR-24-3p is a key factor in the downregulation of TRPM6 expression in the H_2_O_2_-treated NRK-52E cells. The downregulation of TRPM6 expression causes a tubulointerstitial nephropathy in obese T2D rats [11]. However, miR-24-3p has been reported to negatively correlate with the diabetic nephropathy progression [32]. Cell proliferation and fibrosis are inhibited by knockdown of fibroblast growth factor 11, a down-stream target of miR-24-3p. The diabetic nephropathy may not be caused by the alteration of single or few genes.

Extracellular vesicles (EVs) contain various molecules including DNA, mRNA, miRNAs, proteins, and lipids, and are released into extracellular fluid. Urine is a highly useful specimen for biomarker discovery because it can be collected easily using non-invasive techniques. Urinary EVs have been recognized as potential diagnostic biomarkers in renal disease [33]. The content of miR-24-3p in urinary extracellular vesicles from T2D patients with diabetic kidney diseases is higher than that in normal glucose tolerance T2D patients [34]. miR-24-3p may be a novel marker for the T2D patients with diabetic kidney diseases.

### 3.4. Effects of GA, H_2_O_2_, miR-24-3p siRNA on Mg^2+^ Influx

**Figure 4:**
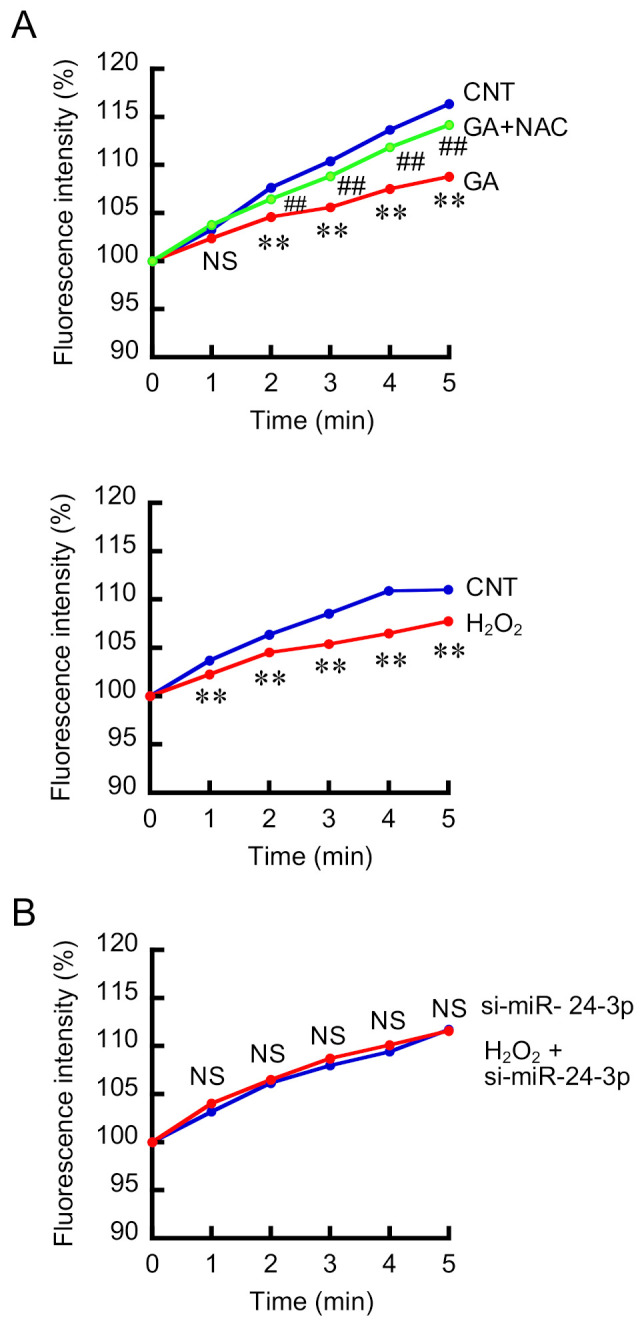
Effects of TRPM6 expression modulators on the elevation of [Mg^2+^]_i_. (**A**) NRK-52E cells were treated in the absence (CNT) or presence of 500 μg/mL GA, 2 mM NAC, and 50 μM H_2_O_2_ for 24 h. [Mg^2+^]_i_ was measured using a fluorescence microplate reader. The extracellular solution was changed to HBSS containing 5 mM MgCl_2_ at 0 min. [Mg^2+^]_i_ was measured using a fluorescence microplate reader and represented as percentage of 0 min. (**B**) The cells transfected with si-Neg or si-miR-24-3p were treated with 50 μM H_2_O_2_ for 24 h. [Mg^2+^]_i_ was represented as percentage of 0 min. *n* = 5–6. ** *p* < 0.01 compared with CNT. ^##^ *p* < 0.01 compared with GA. ^NS^ *p* > 0.05 compared with CNT or si-miR-24-3p.

So far, we reported that Mg^2+^ influx is suppressed by TRPM6 siRNA in NRK-52E cells, indicating that TRPM6 may be functionally expressed [35]. The addition of 5 mM MgCl_2_ in the extracellular solution induced the elevation of fluorescence intensity of Magnesium Green (Figure 4A), which means the elevation of [Mg^2+^]_i_. This effect was suppressed by the treatment with GA, which was rescued by NAC. In addition, the elevation of [Mg^2+^]_i_ was attenuated by H_2_O_2_. These results suggest that Mg^2+^ influx via TRPM6 is inhibited by oxidative stress. The treatment with miR-24-3p siRNA blocked the H_2_O_2_-induced reduction of Mg^2+^ influx (Figure 4B). These effects are similar to those in real-time PCR analysis. We suggest that GA suppresses the expression level of TRPM6 mediated by the elevation of ROS generation and miR-24-3p production, resulting in the inhibition of Mg^2+^ reabsorption in the renal tubular epithelial cells. miR-24-3p may be a novel target for hypomagnesemia therapy in T2D.

## 4. Conclusions

We found that the mRNA level of TRPM6 is decreased by GA and H_2_O_2_ in renal tubular NRK-52E cells. The H_2_O_2_-induced reduction of TRPM6 mRNA and Mg^2+^ influx was rescued by miR-24-3p siRNA. Our data indicate that GA may decrease TRPM6 expression mediated by the elevation of ROS and miR-24-3p in renal tubular epithelial cells of T2D. NRK-52E cells are thought to be a proximal tubular origin because they have similar characteristics including production of collagen and secretion of C-type natriuretic peptide to proximal tubular cells [36]. Therefore, we need further study to clarify whether miR-24-3p is involved in the reduction of TRPM6 expression in the DCT cells.

## Figures and Tables

**Table 1:** Primer pairs for real-time PCR.

Genes	Direction	Sequence (5′→3′)
*TRPM6*	Sense	CTTCTTGGGATACCAAATCAG
	Antisense	GAAACTTTTCCTAGTGTAGCTG
*TRPM7*	Sense	AACCAACACTCTGGAAGAGATCA
	Antisense	TCAGTCAAGTTTTCTCCCACAC
*CNNM2*	Sense	AACACCATCTTCCTCACCAAGT
	Antisense	TCAGCTCTTCCTTAACGAGGTC
*β-Actin*	Sense	CCAACCGTGAAAAGATGACC
	Antisense	CCAGAGGCATACAGGGACAG
*miR-24-3p*	Sense	TGGCTCAGTTCAGCAGGAAC
*let-7d-5p*	Sense	AGAGGTAGTAGGTTGCATAG
*miR-26a-5p*	Sense	TTCAAGTAATCCAGGATAGG
*miR-140-3p*	Sense	ATGGTGTCTTTAGTACCGTC
*miR-143-3p*	Sense	TCTCTACTCCGAGTCTCCCA
*miR-34a*	Sense	CCCGTCACTCTCTACTCCGA
*miR-218-5p*	Sense	AACACGAAAACAAAGTAAGT
*miR-128-3p*	Sense	GGTGTCACAAAGACTCGAAT
*miR-193-3p*	Sense	CTGACCGGTTCCTCTTGTCT

## Data Availability

Not applicable.
